# Comprehensive Analysis of HDAC Family Identifies *HDAC1* as a Prognostic and Immune Infiltration Indicator and *HDAC1*-Related Signature for Prognosis in Glioma

**DOI:** 10.3389/fmolb.2021.720020

**Published:** 2021-09-01

**Authors:** Yuxiang Fan, Xinyu Peng, Yubo Wang, Baoqin Li, Gang Zhao

**Affiliations:** ^1^ Department of Neurosurgery, The First Hospital of Jilin University, Changchun, China; ^2^ Department of Hepatobiliary Pancreatic Surgery, The First Hospital of Jilin University, Changchun, China; ^3^ Department of Spine Surgery, The First Hospital of Jilin University, Changchun, China

**Keywords:** glioma, HDAC1, HDAC family, immune infiltration, prognosis, signature

## Abstract

**Background:** The histone deacetylase (HDAC) family limited accessibility to chromatin containing tumor suppressor genes by removing acetyl groups, which was deemed a path for tumorigenesis. Considering glioma remained one of the most common brain cancers with a dichotomy prognosis and limited therapy responses, HDAC inhibitors were an area of intensive research. However, the expression profiles and prognostic value of the HDACs required more elucidation.

**Methods:** Multiple biomedical databases were incorporated, including ONCOMINE, GEPIA, TCGA, CGGA, GEO, TIMER, cBioPortal, and Metascape, to study expression profiles, prognostic value, immune infiltration, mutation status, and enrichment of HDACs in glioma. STRING and GeneMANIA databases were used to identify *HDAC1*-related molecules. LASSO regression, Cox regression, Kaplan-Meier plot, and receiver operating characteristic (ROC) analyses were performed for *HDAC1*-related signature construction and validation.

**Results:***HDAC1* was significantly overexpressed in glioma, while *HDAC11* was downregulated in glioblastoma. Except for *HDAC 6/9/10*, the HDAC family expression was significantly associated with glioma grade. Most of the HDAC family also correlated with glioma genetic mutations. Higher *HDAC1* expression level predicted more dismal overall survival (OS) (*p* < 0.0001) and disease-free survival (DFS) (*p* < 0.0001), but a higher level of *HDAC11* held more favorable OS (*p* = 2.1e−14) and DFS (*p* = 4.8e−08). *HDAC4* displayed the highest mutation ratio, at 2.6% of the family. The prognostic value of *HDAC1* was validated with ROC achieving 0.70, 0.77, 0.75, and 0.80 as separability for 1-, 3-, 5-, and 10-years OS predictions in glioma, respectively. Moreover, *HDAC1* expression positively correlated with neutrophil (r = 0.60, *p* = 2.88e-47) and CD4^+^ T cell infiltration (r = 0.52, *p* = 3.96e-35) in lower-grade glioma. The final *HDAC1*-related signature comprised of *FKBP3*, *HDAC1* (Hazard Ratio:1.49, 95%Confidence Interval:1.20–1.86), *PHF21A*, *RUNX1T1*, and *RBL1*, and was verified by survival analysis (*p* < 0.0001) and ROC with 0.80, 0.84, 0.83, and 0.88 as separability for 1-, 3-, 5-, and 10-years OS predictions, respectively. The signature was enriched in chromatin binding.

**Conclusion:** HDAC family was of clinical significance for glioma. Most of the HDAC family significantly correlated with the glioma grade, *IDH1* mutation, and *1p/19q* codeletion. *HDAC1* was both a prognostic and immune infiltration indicator and a central component of the *HDAC1*-related signature for precise prognosis prediction in glioma.

## Introduction

Glioma, characterized by its dichotomy prognosis, is one of the most common primary brain tumors in adults (Schiff et al., 2019; Wen et al., 2020). Patients diagnosed with glioblastoma multiforme (GBM, WHO IV grade) typically hold a median survival time of merely 14 months, whereas most cases of low-grade glioma, like pilocytic astrocytoma (WHO I grade), attained clinical cure after surgical resection (Louis et al., 2016; Wen et al., 2020). The malignancy might come from a highly unstable genome and extensive epigenetic deregulation. Since histone modifications were one of the key mechanisms in epigenetics, investigations related to the imbalance between histone acetyltransferases (HATs) and histone deacetylases (HDACs) in glioma are emerging (Kunadis et al., 2021).

Histone acetylation by HATs relaxed chromatin structure and facilitated the transcriptional complex accessing the core histone (Ruijter et al., 2003). By manipulating acetyl groups, HATs and HDACs reached a balance in the regulation of histone structure under physical conditions (Eyüpoglu and Savaskan, 2016). However, a carcinogenic transformation would initiate once the equilibrium was disrupted (Minucci and Pelicci, 2006). One possible mechanism behind the transformation was mediated by the activation of oncogenic genes like *c-Myc* and the repression of the tumor suppressor gene when the HDACs took dominance (Bolden et al., 2006; Nguyen et al., 2020). Therefore, it was of necessity to explore the HDACs in the context of glioma based on their oncogenic properties. The HDAC family so far encompassed four classes, which were further categorized by their dependency on zinc, namely zinc-dependent class I (HDAC1, 2, 3, and 8), class IIa (HDAC4, 5, 7, and 9), class IIb (HDAC 6 and 10), and class IV (HDAC11), and zinc-independent class III (SIRT proteins) (Chen et al., 2020). This study would mainly discuss the HDAC family.

In this research, the HDAC family was systemically analyzed across varieties of databases to evaluate its clinical and prognostic value for glioma. It helped discern the differentially expressed HDAC family genes with significance in glioma compared to normal, as well as the general expression level of each HDAC family member in glioma. The potential ties of the HDAC family to glioma were assessed by detecting the associations between the HDAC family’s expression and glioma grade, and genetic mutations, and by conducting survival analysis on the HDAC family. Genetic alterations, interactive network, and functional enrichment annotations were additionally acquired for the HDAC family. The prognostic gene *HDAC1* and the *HDAC1*-related genes were prepared for the final *HDAC1*-related signature construction. In conclusion, the HDAC family was of prognostic significance and clinical interest for glioma. It exposed the pivotal role of *HDAC1* in glioma, as both an independent prognostic and immune infiltration biomarker and a central component of the *HDAC1*-related signature for precise prognosis prediction.

## Materials and Methods

### Oncomine

The HDAC family member expression profiles in cancers were analyzed in the ONCOMINE database (www.ONCOMINE.org), which enabled public access to resourceful genome-wide cancer microarray data that originated from various studies (Rhodes et al., 2004). Significance thresholds were set as a *p*-value less than 0.01, a fold-change over 2, and gene rank within the top 10%. The detailed information for each study included was listed in Table 1.

**TABLE 1:** Studies on HDAC family in ONCOMINE.

	Study (Glioma vs. Normal)	Fold change	*p*-valueadjusted	*t*-test	Cases (Tumor/Normal)	References
HDAC1	Atypical teratoid tumor	4.597	8.32E-04	5.136	5/4	Pomeroy Nature 2002/01/24
	Anaplastic oligoastrocytoma	2.560	4.41E-04	5.148	4/6	French Cancer Res 2005/12/15
	Glioblastoma	3.131	3.08E-08	14.61	542/10	TCGA 2013/06/03
HDAC2	Desmoplastic medulloblastoma	3.133	1.09E-04	6.188	14/4	Pomeroy Nature 2002/01/24
	Oligodendroglioma	3.235	6.99E-04	8.816	3/7	Shai Oncogene 2003/07/31
	Glioblastoma	−3.683	2.70E-10	−11.1	22/3	Lee Cancer Cell 2006/05/01
	Anaplastic oligoastrocytoma	−4.399	5.95E-04	−5.14	6/4	Bredel Cancer Res 2005/10/01
HDAC4	Glioblastoma	−2.147	1.70E-13	−15.6	80/4	Murat J Clin Oncol 2008/06/20
HDAC5	Anaplastic oligoastrocytoma	−2.092	4.64E-05	−8.11	6/4	Bredel Cancer Res 2005/10/01
	Glioblastoma	−2.378	1.46E-07	−12.2	27/4	Bredel Cancer Res 2005/10/01
	Anaplastic oligodendroglioma	−2.436	0.003	−7.56	3/4	Bredel Cancer Res 2005/10/01
	Glioblastoma	−2.221	6.80E-20	−11.7	81/23	Sun Cancer Cell 2006/04/01
	Glioblastoma	−2.742	3.08E-08	−14.9	542/10	TCGA 2013/06/03
HDAC6	Glioblastoma	3.221	8.88E-13	8.831	81/23	Sun Cancer Cell 2006/04/01
	Anaplastic astrocytoma	2.605	2.34E-06	5.48	19/23	Sun Cancer Cell 2006/04/01
HDAC11	Anaplastic oligoastrocytoma	−4.196	1.09E-05	−9.32	4/6	French Cancer Res 2005/12/15
	Anaplastic oligodendroglioma	−3.927	7.32E-06	−9.90	23/6	French Cancer Res 2005/12/15
	Glioblastoma	−3.539	3.14E-14	−11.0	81/23	Sun Cancer Cell 2006/04/01

### Gene Expression Profiling Interactive Analysis

The mRNA sequencing data of the HDAC family together with its corresponding clinical information of glioma patients were retrieved from GEPIA (http://gepia2.cancer-pku.cn/), a web-based interactive tool providing comprehensive and customizable analyses with The Cancer Genome Atlas (TCGA) and Genotype-Tissue Expression Project (GTEx) RNA sequencing data as resources (Tang et al., 2017). In this study, differential expression analysis comparing 681 gliomas (518 cases of lower-grade glioma, 163 cases of glioblastoma) with 207 normal samples was performed, and survival analysis with Kaplan-Meier (KM) plot and survival heatmap was also included.

### TCGA, Chinese Glioma Genome Atlas, and Gene Expression Omnibus

The glioma cohort in the TCGA research program (https://www.cancer.gov/tcga), including 505 cases of lower-grade glioma (LGG) and 155 cases of GBM RNA sequencing counts data with clinical information, was acquired with the R package “TCGAbiolinks” (Colaprico et al., 2016). The dataset was mainly used as a developing cohort for the construction of the *HDAC1*-related signature. The TCGA developing cohort was then randomly split at a ratio of 3:7 using the R package “caret” for internal validation (Kuhn, 2008).

The glioma RNA-seq dataset “mRNAseq_693” recruited in the CGGA (http://www.cgga.org.cn/) contains 693 glioma samples. It was selected to conduct clinical correlation analysis by linking RNA-seq data with pathological grade and typical mutation statuses, such as *IDH1* mutation and *1p/19q* codeletion. Moreover, the “mRNAseq_693” dataset was employed as verification for previous results from the differential expression analysis and the survival analysis in GEPIA.

The microarray dataset GSE16011 from the Gene Expression Omnibus (GEO) database was gathered as an external validation cohort for the *HDAC1*-related signature (Gravendeel et al., 2009). It consisted of 284 samples, including 117 LGG cases, 156 GBM cases, and normal controls. Patients with clear mutation records were kept.

### cBioPortal

The mutation profiles of each HDAC family member were further analyzed in glioma with help from the cBioPortal for Cancer Genomics (http://cbioportal.org), which was an intuitive web tool for exploring analysis and visualization on cancer genomic data collected from several platforms (Gao et al., 2013). The detailed mutation statuses of the HDAC family in glioma pathological subtypes were also shown.

### Search Tool for the Retrieval of Interacting Genes

STRING (https://string-db.org) pictured both physical and functional protein-protein interaction (PPI) networks based on current knowledge and prediction via systemic co-expression analysis and text-mining of literature (Szklarczyk et al., 2017). The PPI network analysis was used to identify genes associated with the HDAC family members and *HDAC1*. The HDAC family PPI network was visualized by the Cytoscape app (version 3.7.2).

### Metascape

Metascape (http://metascape.org) served as a web-based portal mainly for gene annotation, interactome analysis, and functional enrichment analysis (Zhou et al., 2019). The HDAC family and the *HDAC1*-related gene signature were uploaded to query for the functional interpretation of those genes in fixed combination, thereby enlightening future investigation.

### Tumor Immune Estimation Resource

TIMER database (http://timer.cistrome.org) was introduced to measure immune infiltration in the tumor microenvironment and attain a better comprehension of tumor-immune interactions (Li et al., 2020). Considering feasibility, “immune privilege” in the central nervous system (CNS), and loading capacity for each immune infiltration estimation analysis, only a fraction of typical infiltrated immune cells in glioma, including neutrophils, macrophages, myeloid-derived suppressive cells (MDSCs), CD4^+^ and CD8^+^ T cells, and regulatory T cells (Tregs), was filtered out to check for potential links with *HDAC1* expression.

### GeneMANIA

Except for STRING, GeneMANIA (http://www.genemania.org) was also used for identifying possible *HDAC1*-related genes as candidate genes for further *HDAC1*-related gene signature construction (Franz et al., 2018). The network depicting genes functionally close to *HDAC1* was illustrated by recognizing patterns of gene co-annotation in the Gene Ontology or by using enrichment analysis.

### Prognostic *HDAC1*-Related Gene Signature Development and Validation

The time-dependent receiver operating characteristic (ROC) analysis was conducted to test the predictive value for *HDAC1*, *HDAC2*, and *HDAC11*. Further, the *HDAC1*-related genes derived from GeneMANIA and STRING were regarded as predictive candidates for the *HDAC1*-related signature development. LASSO regression, univariate, and multivariate Cox regression analyses were consecutively applied to calculate the prognostic risk. Only genes with prognostic value and significance when fitting into the signature would be selected. The coefficients in the calculated regression model for each included gene were utilized to calculate the risk score, $Riskscore=\overset{n}{\underset{i=1}{\sum }}\;\beta_{i}\times gene_{i}$, where β represented the coefficient. The predictive nature of the signature components was represented by Hazard Ratio (HR) and 95% Confidence Interval (95%CI). The ROC analysis and survival analysis were performed for the signature discriminating capacity evaluation in the TCGA developing cohort and the GEO validation cohort.

### Statistical Analysis

The statistical analyses and graphs associated with *HDAC1*-related gene signature development and validation were achieved in R 3.6.2 (R Core Team, 2019) and RStudio (version 1.1.463). The Least Absolute Shrinkage and Selection Operator (LASSO) regression analysis, Cox regression analysis, and Kaplan-Meier survival analysis along with proportional hazards (PH) test were performed with the R package “survminer” and the R package “survival.” Wilcoxon test and Kruskal Wallis test were used for statistical comparisons. In this study, a *p*-value less than 0.05 was regarded as statistically significant, and the *p*-values were adjusted with the “BH” method if involved with multiple hypothesis testing.

## Results

### Differential Expression Analysis of HDAC Family Genes in Glioma

**FIGURE 1:**
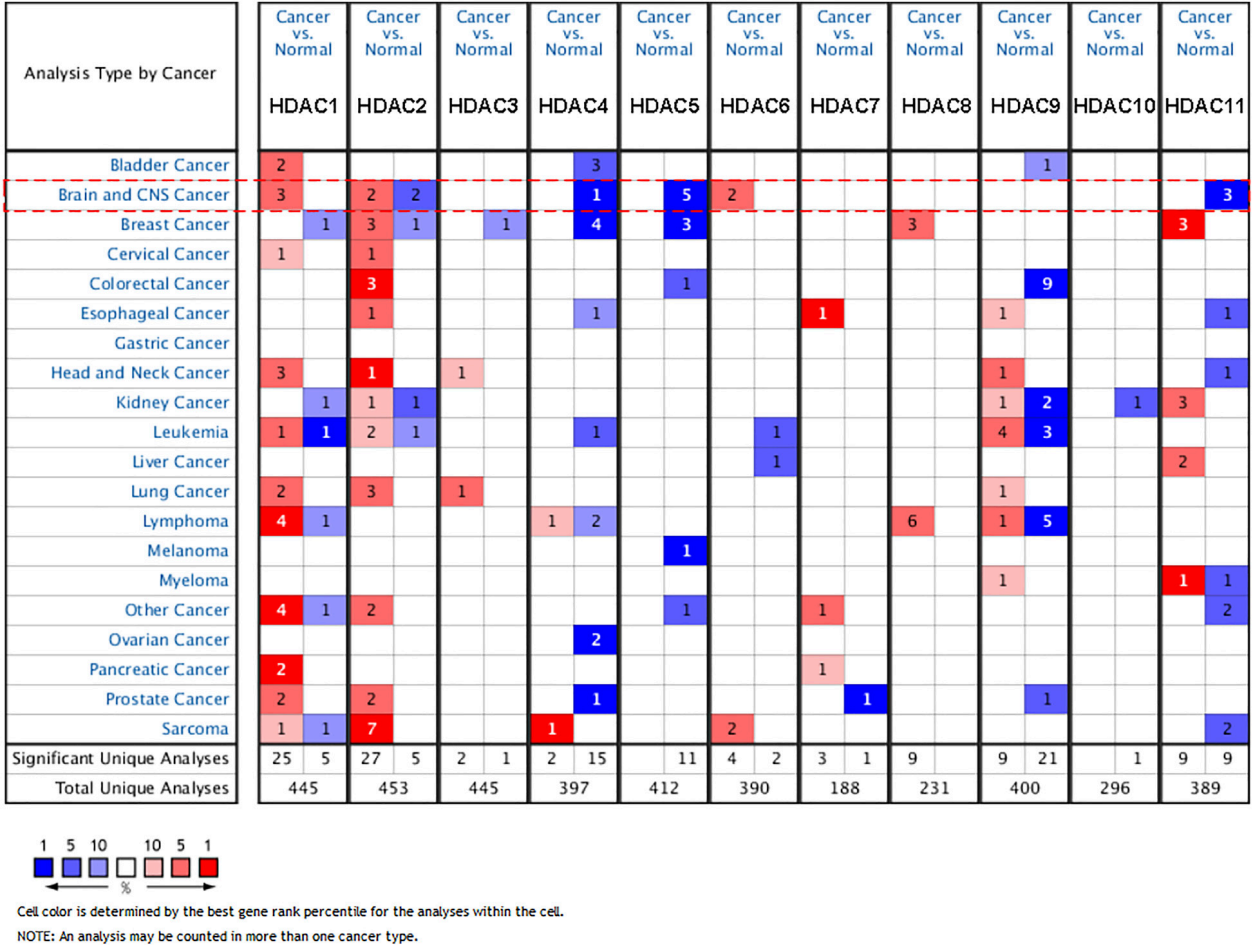
Expression microarray of HDAC family across varieties of cancers and corresponding control groups in ONCOMINE. The number in cells indicated the significant datasets showing the differentially expressed genes of the HDAC family. The red color in the cell was for upregulation, and the blue color was for downregulation. The student’s t-test was used to compare the different transcriptional values. The threshold was set as *p*-value < 0.01, fold-change = 2, gene rank < 10%.

The transcriptional profiles of the HDAC family in brain and CNS cancers were investigated in the ONCOMINE database (Figure 1). It revealed that *HDAC1* and *HDAC6* were significantly over-expressed and within the top 5% gene rank in glioma compared to the normal group, which was evidenced by three and two studies, respectively. In contrast, under-expressed *HDAC4*, *HDAC5*, and *HDAC11* ranking within the top 1% gene rank were observed in glioma. It showed that two studies supported significantly overexpressed *HDAC2* in CNS cancer, but an equal number of studies concluded otherwise. The evidence with differential significance and top gene ranking for the rest of the HDAC family members remained scarce. The detailed study information regarding the HDAC family in ONCOMINE was summarized in Table 1.

*HDAC1* mRNA over-expression in anaplastic oligoastrocytoma (*n* = 4) reached a fold change (FC) of 2.56 compared with the normal tissues (*n* = 6) (*p* = 4.41E-04) (French et al., 2005). In GBM samples (*n* = 542) from the TCGA cohort, *HDAC1* showed 3.13-fold increase of expression (*p* = 3.08E-08). Over-expressed *HDAC6* showed FCs of 3.22 and 2.61 for GBM (*n* = 81) (*p* = 8.88E-13) and anaplastic astrocytoma (*n* = 19) (*p* = 2.34E-06) groups, respectively (Sun et al., 2006).

Moreover, it was detected that the *HDAC4* expression in GBM (*n* = 80) was 2.15-fold lower than the control group (*n* = 4) (*p* = 1.70E-13) (Murat et al., 2008). The comparisons of *HDAC5* expression between anaplastic oligoastrocytoma (*n* = 6), anaplastic oligodendroglioma (*n* = 3), GBM (*n* = 27), and normal (*n* = 4) exposed a 2.10-fold (*p* = 4.64E-05), 2.44-fold (*p* = 0.003), 2.38-fold (*p* = 1.46E-07) decrease, respectively (Bredel et al., 2005). *HDAC5* mRNA level in GBM (*n* = 81) was down-regulated with a FC of 2.22 in Sun’s 2006 study (*p* = 6.80E-20), and a fold change of 2.74 in the GBM cohort (*n* = 542) from TCGA (*p* = 3.08E-08) (Sun et al., 2006). *HDAC11* displayed 4.20-fold downregulation in anaplastic oligoastrocytoma (*n* = 4) (*p* = 1.09E-05), and 3.93-fold downregulation in anaplastic oligodendroglioma (*n* = 23) in the French 2005 study (*p* = 7.32E-06) (French et al., 2005). Similarly, *HDAC11* was found to transcriptionally decrease with a FC of 3.54 in GBM (*n* = 81) (*p* = 3.14E-14) (Sun et al., 2006).

The studies focusing on *HDAC2* were of interest in ONCOMINE. One medulloblastoma study was filtered out. However, the rest of the three studies led to conflicting conclusions. As oligodendroglioma (*n* = 3) was compared with normal (*n* = 7) in Shai’s 2003 study, *HDAC2* was upregulated and showed a fold change of 3.24 (*p* = 6.99E-04). The other two studies found that *HDAC2* significantly under-expressed in anaplastic oligoastrocytoma (*n* = 6) with a fold change of 4.40 (*p* = 5.95E-04), and in GBM (*n* = 22) with a fold change of 3.68 (*p* = 2.70E-10).

**FIGURE 2:**
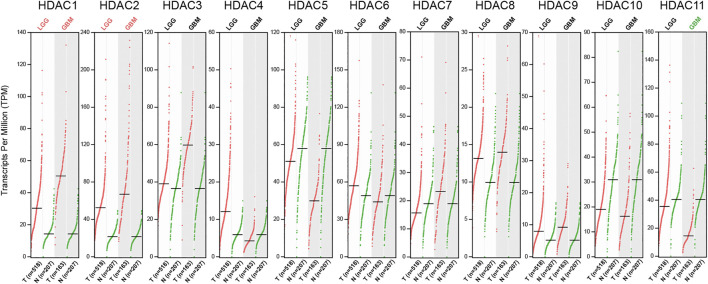
RNA-seq data of HDAC family in LGG and GBM accessed from GEPIA. The Scatter plots showed expression comparisons of the HDAC family between the tumor and the normal groups. The statistically significant comparison with |Log_2_FC| over 1 was marked either in red (upregulation) or green (downregulation) (q < 0.01).

To fully investigate the HDAC family expression in glioma, the GEPIA database, which incorporated the GTEx and the TCGA data, was explored for verification. In the differentially expressed gene analysis, each HDAC family member was studied in both LGG (*n* = 518) and GBM (*n* = 163) subsets with thresholds of |Log_2_FC| over 1 and q-value less than 0.01. Of the entire HDAC family, only *HDAC1*, *HDAC2*, and *HDAC11* were tested to have more than a 2-fold alteration in expression with significance compared to normal tissue (Figure 2). *HDAC1* and *HDAC2* mRNA level was upregulated in both LGG and GBM, which was over 2-fold higher than the normal, whereas *HDAC11* expression level was more than 2-fold lower in the GBM group only.

### Links Between HDAC Family and Glioma Grade, and Genetic Mutations

**FIGURE 3:**
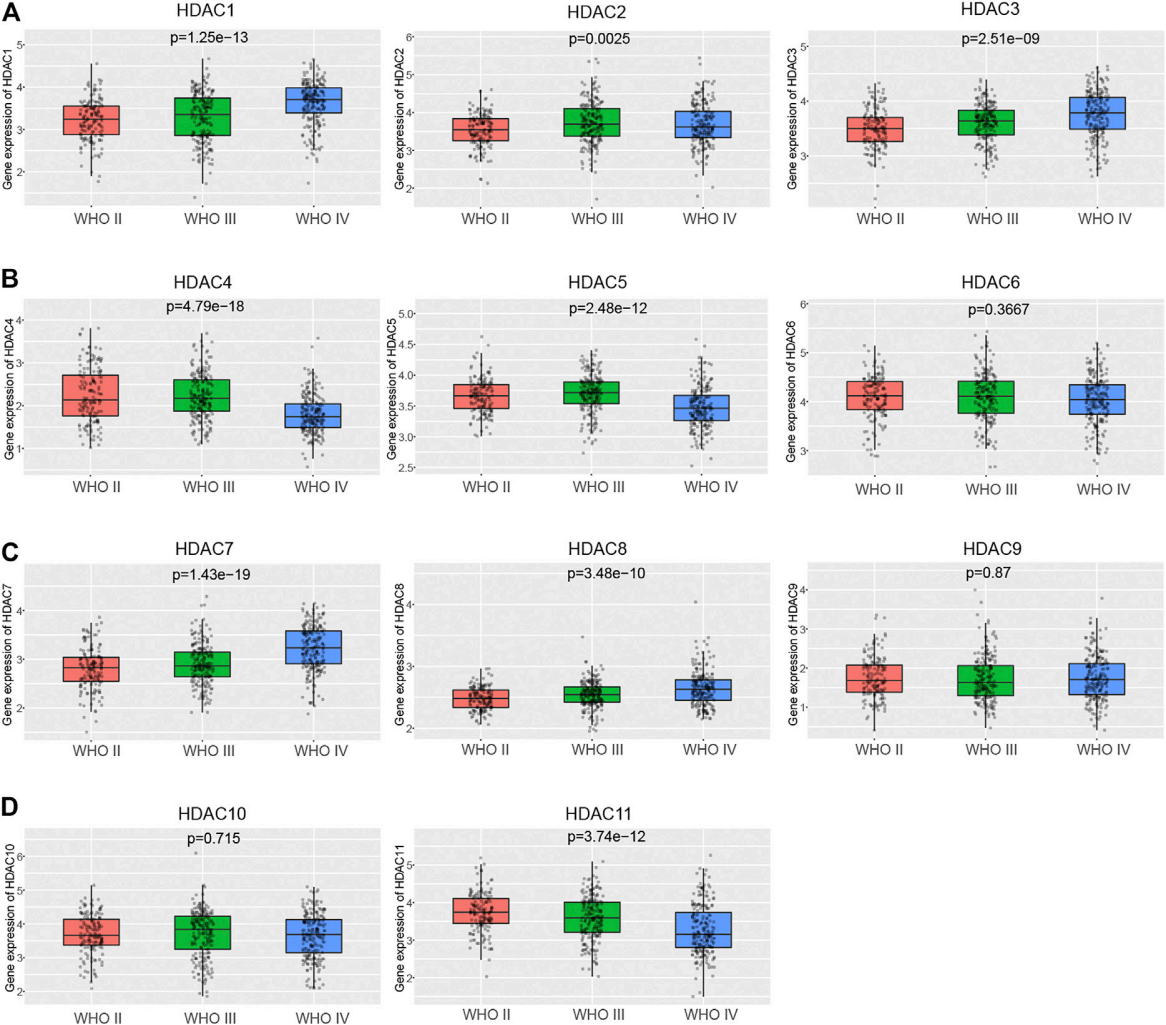
Links between HDAC family and glioma grade in CGGA. **(A–D)** The box plots depicted that the expression of the HDAC family changed with glioma grade.

The relationship between the HDAC family and clinical features was studied. It was of priority to check whether the HDAC family’s expression altered with glioma pathological grade using the glioma cohort in CGGA. Except for *HDAC6* (*p* = 0.367), *HDAC9* (*p* = 0.870), and *HDAC10* (*p* = 0.715), the majority of HDAC family expression levels correlated significantly with glioma grade (Figure 3). Specifically, the mRNA level of *HDAC1* (*p* = 1.25e−13), *HDAC3* (*p* = 2.51e−09), *HDAC7* (*p* = 1.43e−19), and *HDAC8* (*p* = 3.48e−10) showed an increasing trend with glioma grade progressing (Figures 3A,C). On the contrary, *HDAC11* (*p* = 3.74e−12) expression was negatively associated with glioma grade (Figure 3D). *HDAC2* (*p* = 0.0025) exhibited the highest expression level in the WHO III group but the lowest one in the WHO II group (Figure 3A). It also revealed that *HDAC4* (*p* = 4.79e−18) and *HDAC5* (*p* = 2.48e−12) expressed the most in the WHO III group but the least in the WHO IV group (Figure 3B).

**FIGURE 4:**
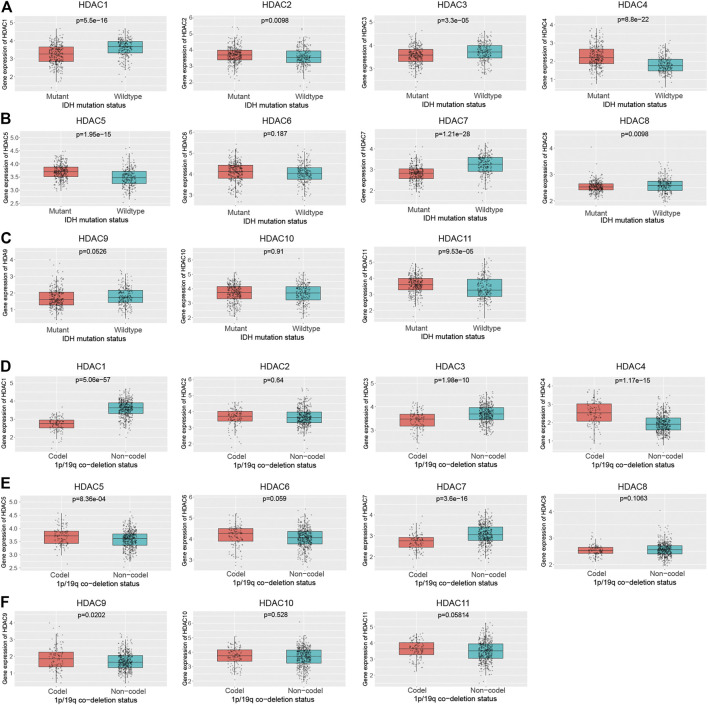
Correlation between HDAC family and glioma genetic mutation in CCGA. **(A–F)** The box plots showed that the expression of the HDAC family varied according to the glioma mutations.

Since the prognosis of glioma mainly depends on the pathological grade and genetic mutations, among which isocitrate dehydrogenase 1 (*IDH1*) and *1p/19q* status represented primary concern, further investigation was conducted in an attempt to research relations between HDAC family and *IDH1* and *1p/19q* based on the CGGA glioma cohort. The HDAC family, other than *HDAC6* (*p* = 0.187), *HDAC9* (*p* = 0.0526), and *HDAC10* (*p* = 0.91), are differentially expressed in the *IDH1* mutant and *IDH1* wildtype group with significance (Figure 4). As shown in the figure, *HDAC1* (*p* = 5.5e−16), *HDAC3* (*p* = 3.3e−05), *HDAC7* (*p* = 1.21e−28), and *HDAC8* (*p* = 0.0098) expression levels were significantly higher in the *IDH1* wildtype than the mutant (Figures 4A–C). However, *HDAC2* (*p* = 0.0098), *HDAC4* (*p* = 8.80e−22), *HDAC5* (*p* = 1.95e−15), and *HDAC11* (*p* = 9.53e−05) significantly expressed more transcripts in the *IDH1* mutant group compared to the wildtype (Figures 4A–C).

The HDAC family members were also characterized by *1p/19q* mutation (Figure 4). It turned out to have the same expression levels regarding the *1p/19q* co-deletion statuses in *HDAC2* (*p* = 0.64), *HDAC6* (*p* = 0.059), *HDAC8* (*p* = 0.106), and *HDAC10* (*p* = 0.528), and *HDAC11* (*p* = 0.0581) (Figures 4D–F). *HDAC1* (*p* = 5.06e−57), *HDAC3* (*p* = 1.98e−10), and *HDAC7* (*p* = 3.6e−16) showed higher expression levels in the *1p/19q* non-codeletion group in contrast to the codeletion group (Figures 4D,E). While *HDAC4* (*p* = 1.17e−15), *HDAC5* (*p* = 8.36e-04), and *HDAC9* (*p* = 0.0202) showed higher expression levels in the *1p/19q* codeletion group (Figures 4D–F).

### Prognostic Characteristics of HDAC Family Genes in Glioma

Apart from the links with glioma grade and genetic mutations, survival analysis based on GEPIA (*n* = 676) and CGGA (*n* = 404) data enabled the prognostic evaluation for the clinical value of the HDAC family. The survival analysis mainly focused on overall survival (OS) and disease-free survival (DFS).

**FIGURE 5:**
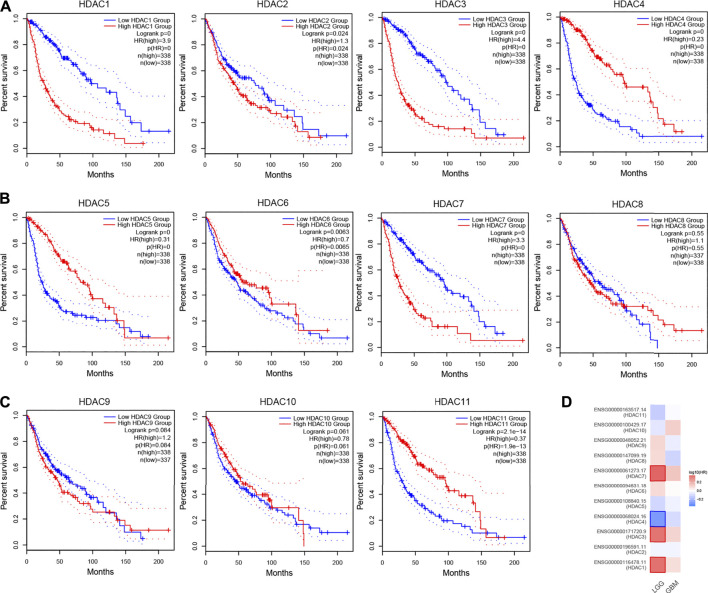
Prognostic evaluation (OS) of HDAC family in glioma based on GEPIA. **(A**–**C)** The KM survival curves showed distinct survival possibility predicted by varying expression of HDAC family. **(D)** The heatmap was used for the link between the HDAC family members’ expression and the OS of LGG and GBM patients.

The OS analysis based on GEPIA revealed that the glioma patients with high expression levels of *HDAC1* (HR:3.9, *p* < 0.0001), *HDAC2* (HR:1.3, *p* = 0.024), *HDAC3* (HR:4.4, *p* < 0.0001), and *HDAC7* (HR:3.3, *p* < 0.0001) would face with more risks compared to the ones with low expression of these genes (Figures 5A,B). However, the patients would reap OS benefits if they expressed high levels of *HDAC4* (HR:0.23, *p* < 0.0001), *HDAC5* (HR:0.31, *p* < 0.0001), *HDAC6* (HR:0.7, *p* = 0.0063), and *HDAC11* (HR:0.37, *p* = 2.1e−14) (Figures 5A–C). The transcriptional levels of *HDAC8* (*p* = 0.55), *HDAC9* (*p* = 0.084), and *HDAC10* (*p* = 0.061) imposed less influence on OS than the other HDAC family members (Figures 5B,C). Further research was performed to detect the impact that the HDAC family exerted on the OS of patients with LGG and GBM, in which *HDAC1*, *HDAC3*, and *HDAC7* remained the top three risk genes, whereas *HDAC4* was a remarkably protective gene (Figure 5D).

**FIGURE 6:**
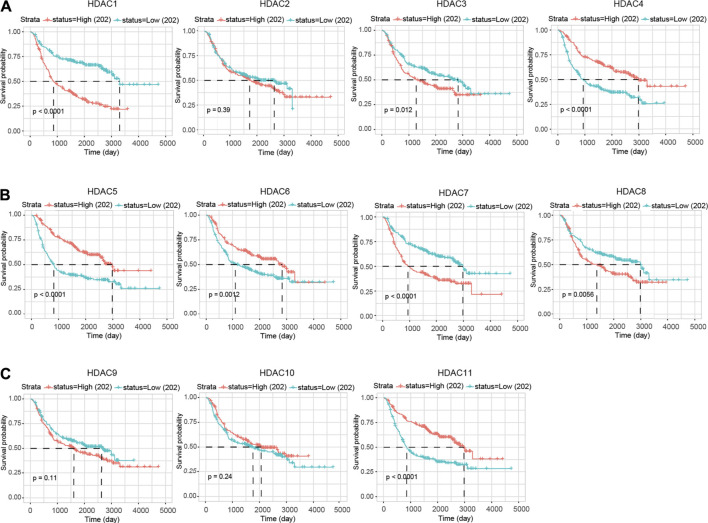
Prognostic feature (OS) of HDAC family in glioma based on CGGA. **(A–C)** The KM survival analysis was performed for verification regarding the prediction efficiency of the HDAC family.

The survival analysis was also applied in the CGGA cohort to validate the prognostic value of the HDAC family. Only *HDAC1* (*p* < 0.0001), *HDAC3* (*p* = 0.012), *HDAC7* (*p* < 0.0001), and *HDAC8* (*p* = 0.0056) were tested with significance for a favorable prognosis if modestly expressed (Figures 6A,B). In addition, overexpressed *HDAC4* (*p* < 0.0001), *HDAC5* (*p* < 0.0001), *HDAC6* (*p* = 0.0012), and *HDAC11* (*p* < 0.0001) enabled the patients to survive longer (Figures 6A–C). The other HDACs showed neither survival benefits nor risks.

**FIGURE 7:**
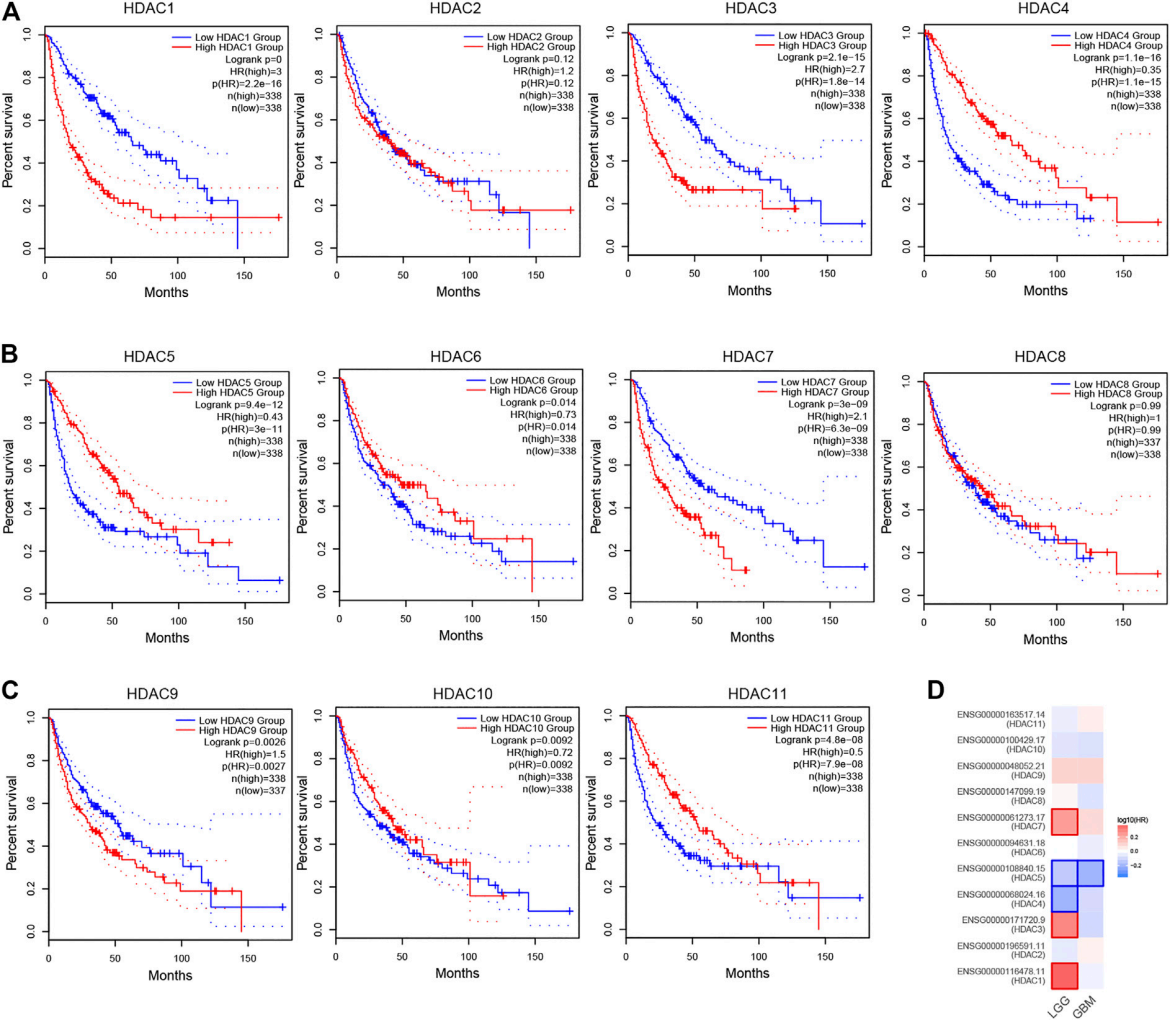
Prognostic value (DFS) of HDAC family in glioma based on GEPIA. **(A**–**C)** The KM survival curves revealed contrasting survival possibility predicted by varying expression of HDAC family. **(D)** The heatmap was used for detecting the relations between the HDAC family members’ expression and the DFS of LGG and GBM patients.

The DFS-oriented study using GEPIA data showed that more transcripts of *HDAC1* (HR:3.0, *p* < 0.0001), *HDAC3* (HR:2.7, *p* = 2.1e−15), *HDAC7* (HR:2.1, *p* = 3e−09), and *HDAC9* (HR:1.5, *p* = 0.0026) accompanied with less DFS probabilities in glioma patients (Figures 7A–C). However, extended DFS would be observed in patients with high expression of *HDAC4* (HR:0.35, *p* = 1.1e−16), *HDAC5* (HR:0.43, *p* = 9.4e−12), *HDAC6* (HR:0.73, *p* = 0.014), *HDAC10* (HR:0.72, *p* = 0.0092), and *HDAC11* (HR:0.5, *p* = 4.8e−08). The rest of the HDAC family displayed no risks or benefits for DFS in glioma patients (Figures 7A–C). Similarly, *HDAC1*, *HDAC3*, and *HDAC7* accounted for the major risk factors for LGG, but *HDAC4* and *HDAC5* enhanced DFS probabilities (Figure 7D).

### Genetic Mutations, Interactive Network, and Functional Enrichment Analysis of HDAC Family

**FIGURE 8:**
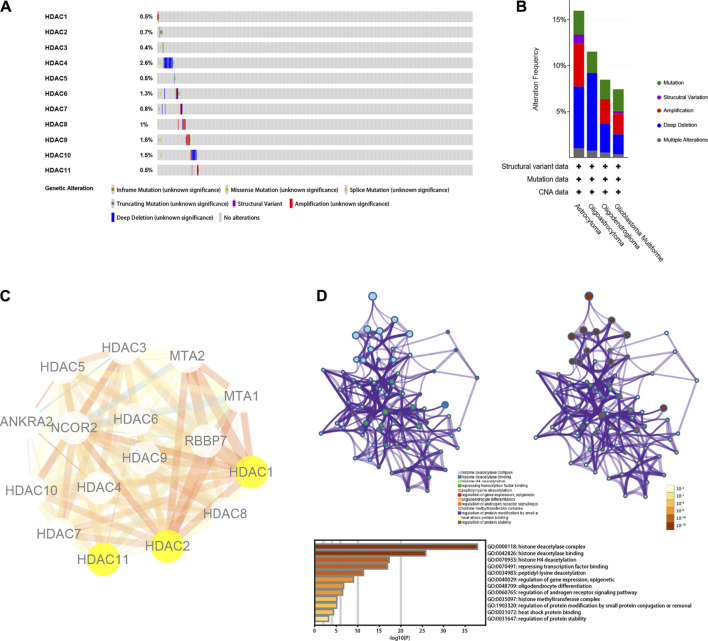
Genetic mutation, interactive network, and functional enrichment of HDAC family. **(A**,**B)** Summary for genetic alterations of HDAC family in glioma. **(C)** Protein-protein interactive network of HDAC family by STRING. The size of the edges in the network was set to change according to the co-expression value among the nodes. The color of the edges in the network altered with the combined scores from the low to the high, which were all the evidence scores (including transferred scores). **(D)** Functional enrichment plot and annotation by Metascape.

The genetic mutations of the HDAC family were analyzed with the TCGA data available at the cBioPortal database. Each of the HDAC family members harbored genetic mutations, in which *HDAC4* was the most prominent with a mutation ratio of 2.6% (Figure 8A). It was followed by the mutation ratio of *HDAC9*, *HDAC10*, and *HDAC6* being 1.5, 1.5, and 1.3%, respectively (Figure 8A). Interestingly, the mutation ratio of *HDAC1* and *HDAC11* were the same at 0.5% (Figure 8A). In terms of mutation statuses in the detailed glioma subtypes, astrocytoma and GBM shared a similar distribution of mutation patterns. However, oligoastrocytoma only showed three kinds of genetic alterations, “mutation,” “deep deletion,” and “multiple alterations,” and oligodendroglioma harbored one more type compared to oligoastrocytoma, “amplification” (Figure 8B).

Further, interaction analysis was performed in the HDAC family using the STRING web tool. Beyond the HDAC family were *MTA1*, *MTA2*, *RBBP7*, *NCOR2*, and *ANKRA2* that were incorporated in the PPI network (Figure 8C). The functional enrichment annotated the interactive network mainly as “histone deacetylation” (Figure 8D). Nevertheless, enrichment results, such as “oligodendrocyte differentiation,” “histone methyltransferase,” and “heat shock protein binding,” were also noticeable.

### Associate Between Prognostic *HDAC1* and Immune Infiltration in Glioma

**FIGURE 9:**
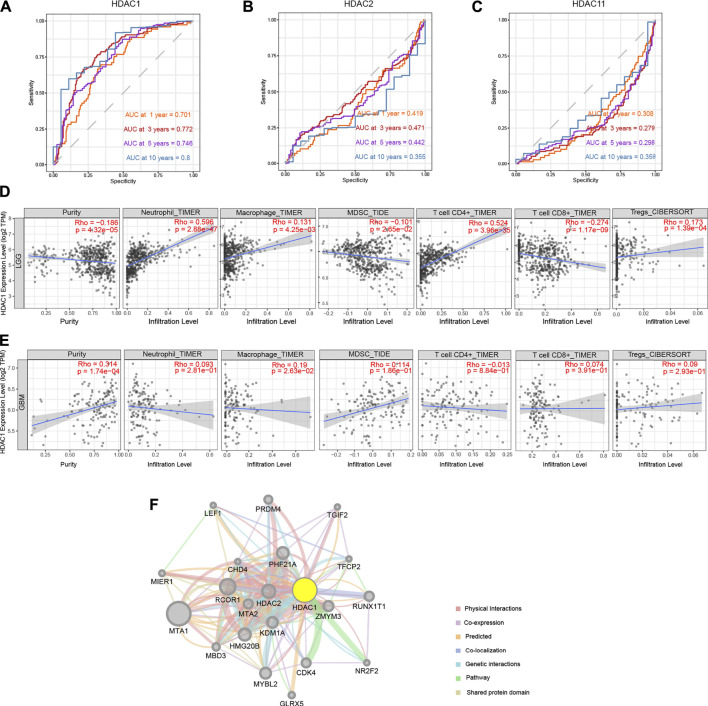
Correlation between prognostic gene *HDAC1* and immune infiltration, and related proteins. **(A**–**C)** Time-dependent ROC curve on *HDAC1*, *HDAC2*, and *HDAC11* checking for OS prediction accuracy at 1-, 3-, 5-, and 10-years time points. **(D**,**E)** Correlation between *HDAC1* and neutrophil, macrophage, MDSC, CD4^+^ T cell, CD8^+^ T cell, and Treg infiltration in LGG and GBM. **(F)** Identification of *HDAC1*-related gene network by GeneMANIA.

Based on the results summarized in Table 2, *HDAC1*, *HDAC2*, and *HDAC11* were selected as the predictive genes with significant clinical value considering their significant and consistent performance in the previous analyses. ROC analysis was applied to assess the discrimination efficiency of the three genes in the TCGA glioma cohort. The separability of the *HDAC1* turned out to be valid, reaching 0.70, 0.77, 0.75, and 0.80 for 1-, 3,- 5-, and 10-years OS predictions in glioma, respectively (Figure 9A). However, the area under the ROC curve (AUC) of the *HDAC2* and the *HDAC11* was calculated with no value exceeding 0.5 throughout the time points in the study (Figures 9B,C).

**TABLE 2:** Summary of the HDAC family analysis overall result significance *p*-value.

	HDAC1	HDAC2	HDAC3	HDAC4	HDAC5	HDAC6	HDAC7	HDAC8	HDAC9	HDAC10	HDAC11
**Gene expression** (glioma vs. control)											
ONCOMINE	3.08e-08	2.70e-10	—	1.70e-13	6.80e-20	8.88e-13	—	—	—	—	3.14e-14
GEPIA (|Log2FC|>1)	<0.0100	<0.0100	>0.0100	>0.0100	>0.0100	>0.0100	>0.0100	>0.0100	>0.0100	>0.0100	(GBM) < 0.01
**Clinical trait correlation**											
CGGA (WHO grade II - III - IV)	1.25e−13	0.0025	2.51e−09	4.79e−18	2.48e−12	0.3670	1.43e−19	3.48e−10	0.8700	0.7150	3.74e−12
CGGA (*IDH1* mutation - *IDH1* wildtype)	5.50e−16	0.0098	3.30e−05	8.80e−22	1.95e−15	0.1870	1.21e−28	0.0098	0.0526	0.9100	9.53e−05
CGGA (*1p/19q* codeletion - non-codeletion)	5.06e−57	0.6400	1.98e−10	1.17e−15	8.36e-04	0.0590	3.60e−16	0.1063	0.0202	0.5280	0.0580
**Survival analysis** (high-expression vs. low-expression)											
GEPIA (Overall survival)	<0.0001	0.0240	<0.0001	<0.0001	<0.0001	0.0063	<0.0001	0.5500	0.0840	0.0610	2.10e−14
CGGA (Overall survival)	<0.0001	0.3900	0.01200	<0.0001	<0.0001	0.0012	<0.0001	0.0056	0.1100	0.2400	<0.0001
GEPIA (Disease Free survival)	<0.0001	0.1200	2.10e−15	1.10e−16	9.40e−12	0.01400	3.00e−09	0.9900	0.0026	0.0092	4.80e−08

Immune infiltration presumably involved with glioma progression and prognosis. Since *HDAC1* was filtered out to be the prognostic gene for glioma, the association between the immune infiltration and the *HDAC1* expression was analyzed by the TIMER database. Considering the limited immune cells in the CNS system due to the BBB, neutrophils, macrophages, MDSCs, CD4^+^ T cells, CD8^+^ T cells, and Tregs were regarded as the major infiltrated immune cell types in glioma for the evaluation. The correlation test was categorized by LGG and GBM.

It revealed that each result of the immune correlation analysis was of significance (*p* < 0.0 1) but a relatively strong relation (r > 0.5) was limited either in LGG or GBM (Figures 9D,E). Intriguingly, *HDAC1* mRNA level was relatively closely and positively tied to neutrophil infiltration in the LGG group (r = 0.60, *p* = 2.88e-47) (Figure 9D). The *HDAC1* expression also positively associated with CD4^+^ T cell infiltration (r = 0.52, *p* = 3.96e-35) (Figure 9D) in the LGG group. The negative relation between CD8^+^ T cell infiltration and *HDAC1* expression level was noticeable (r = -0.27, *p* = 1.17e-09) (Figure 9D). In the GBM group, the strongest relationship was between macrophage and *HDAC1* expression (r = 0.19, *p* = 2.81e-01) (Figure 9E). Additionally, *HDAC1* was used to explore the potential *HDAC1*-related genes in the GeneMANIA (Figure 9F). The entire *HDAC1*-centered interactive network (Figure 9F) together with supplementary genes acquired from the STRING in the same way as the GeneMANIA, the candidate risk genes for *HDAC1*-related signature construction, were thus recruited. The *HDAC1*-related genes were listed in Supplementary Table S1.

### Development and Validation of *HDAC1*-Related Gene Signature

**FIGURE 10:**
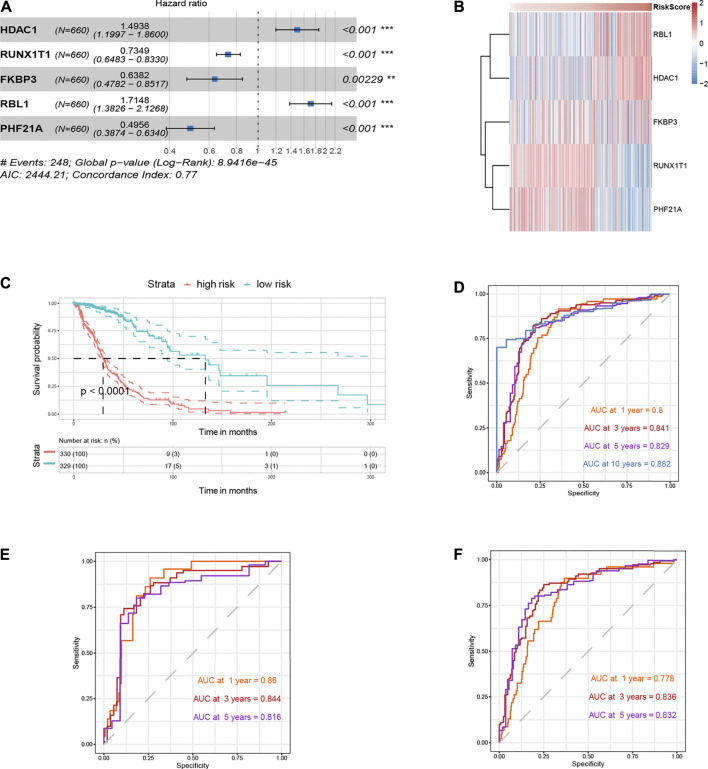
*HDAC1*-related gene signature construction and internal validation. **(A)** Forest plot for the *HDAC1*-related gene signature. **(B)** Heatmap for expression profiles of the signature components in the TCGA glioma cohort. **(C)** Survival curve for survival possibility test between high- and low-risk group according to the signature risk score in the TCGA cohort. **(D)** Time-dependent ROC curve for the separability test of the *HDAC1*-related signature at 1-, 3-, 5-, and 10-years time points (OS) using the TCGA developing cohort. **(E)** Time-dependent ROC curve for the *HDAC1*-related signature using the TCGA training set. **(F)** Time-dependent ROC curve for the *HDAC1*-related signature using the TCGA testing set.

Fifteen genes were screened out of the initial *HDAC1*-related genes through LASSO regression analysis (Supplementary Figure S1). Univariate and multivariate Cox regression analyses were then performed for the signature development (Supplementary Table S2). The *HDAC1*-related gene signature was ultimately constructed in the TCGA cohort and comprised of five promising prognostic genes: *HDAC1* (HR:1.4938, 95%CI:1.1997–1.8600), *RUNX1T1* (HR:0.7349, 95%CI:0.6483–0.8330), *FKBP3* (HR:0.6382, 95%CI:0.4782–0.8517), *RBL1* (HR:1.7148, 95%CI:1.3826–2.1268), and *PHF21A* (HR:0.4956, 95%CI:0.3874–0.6340) (Figure 10A). The signature model satisfied the proportional hazards (PH) assumption (Supplementary Figure S2). And it was also adjusted and tested with other prognostic factors, including gender, grade, *IDH1* mutation, and *1p/19q* codeletion, to be an independent prognostic indicator (Riskscore, HR:1.0935, 95%CI:1.0045–1.1904, *p* = 0.039) (Supplementary Figure S3). The signature risk score was calculated as follows: Riskscore = 0.4013×*HDAC1*-0.3081×*RUNX1T1*-0.4491×*FKBP3*+0.5393×*RBL1*-0.7020×*PHF21A*.

The expression profiles of the *HDAC1*-related signature components were investigated in the TCGA glioma cohort (Figure 10B). *HDAC1* and *RBL1* were upregulated with the increasing risk score, while the expression levels of *FKBP3*, *RUNX1T1*, and *PHF21A* reversed (Figure 10B). To validate the prognostic efficiency, the signature was tested by survival analysis and ROC curve in the TCGA developing cohort. It showed that the high-risk group which scored high according to the equation held poorer survival probabilities than the low-risk (*p* < 0.0001) (Figure 10C). Additionally, the discrimination of the signature was measured by the AUC being 0.800, 0.841, 0.829, and 0.882 for 1-, 3-, 5- and 10-years OS, respectively (Figure 10D).

**FIGURE 11:**
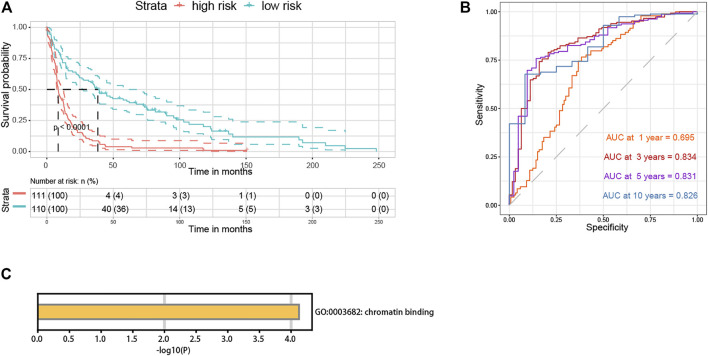
*HDAC1*-related gene signature external validation and enrichment analysis. **(A)** Survival curve for survival possibility test between high- and low-risk group according to the signature risk score in the GEO cohort. **(B)** Time-dependent ROC curve for the separability test of the *HDAC1*-related signature at 1-, 3-, 5-, and 10-years time points (OS) using the GEO validation cohort. **(C)** Functional enrichment analysis of the *HDAC1*-related signature.

The signature was also validated internally and externally. As for internal validation, the TCGA developing cohort was randomly divided into two sets, one for training containing 30% of the cohort cases, and the other as the testing set. It classified the data with the 1-year OS AUC being 0.860, the 3-years OS AUC being 0.844, and the 5-years OS AUC being 0.816 in the training set (Figure 10E). Moreover, the 1-year OS AUC being 0.778, the 3-years OS AUC being 0.836, and the 5-years OS AUC being 0.832 were measured in the testing set (Figure 10F). In the GEO validation cohort used for external validation, the high-risk group consistently had a higher survival risk compared to the low one (*p* < 0.0001) (Figure 11A). And the discrimination of the signature was confirmed in the GEO cohort regarding being 0.695, 0.834, 0.831, and 0.826 for 1-, 3-, 5-, and 10-years OS, respectively (Figure 11B). The signature was annotated as “chromatin binding” by the functional enrichment analysis in Metascape (Figure 11C). The clinical baseline information of the TCGA developing cohort and the GEO validation cohort were listed in Supplementary Table S3.

## Discussion

Differentially expressed HDAC family members were identified with significance in ONCOMINE, namely the upregulated *HDAC1* and *HDAC6*, as well as the downregulated *HDAC4*, *HDAC5,* and *HDAC11* in glioma (Figure 1). After ruling out the unrelated medulloblastoma study, the contradictory expression profiles in the three studies on *HDAC2* resulted in a perplexing conclusion, of which one indicated overexpression while the other two opposed. However, the study size of the three studies was too limited to conclude firmly regarding *HDAC2* expression level in glioma. The Lee 2006 study recruiting 22 cases of GBM which was larger than the size of the other two *HDAC2* studies might suggest that *HDAC2* was under-expressed in glioma (Table 1).

Further validation of the HDAC family differential expression was conducted in GEPIA. Only the comparison with a change over 2-fold and q-value less than 0.01 was marked in the exhibition (Figure 2). The *HDAC1* and *HDAC2* comparisons in glioma, and the *HDAC11* comparison in GBM, were remarkable for their significantly contrasting expression. The remaining groups were neither of significance nor holding differential changes exceeding 2-fold. A previous study composed of 20 low-grade and 23 high-grade glioma patients concluded that class II and IV HDACs expressed less in GBM compared to low-grade glioma and normal tissue (Lucio-Eterovic et al., 2008; Williams et al., 2017). However, it seemed too bold and assertive to firmly conclude with such a limited sample size. Besides, the single-centered data might be insufficient and unpersuasive since the differential expression results from multiple sources in this study only agree with a few genes to be further analyzed. Considering the findings from ONCOMINE, GEPIA, and CGGA, *HDAC1*, *HDAC2*, and *HDAC11* were thus verified for their differential expression in glioma (Figures 1, 2, 3; Table 2).

It was assumed that the differentially expressed HDAC family might contribute to the clinical and genetic features of glioma. The investigation on the potential links between the HDAC family and the pathological grade, and the genetic alterations of glioma, revealed that the expression levels of *HDAC1*, *HDAC2*, and *HDAC11* significantly altered with the glioma grade (*HDAC1*, *p* = 1.25e−13; *HDAC2*, *p* = 0.0025; *HDAC11*, *p* = 3.74e−12) and the *IDH1* mutation status (*HDAC1*, *p* = 5.5e−16; *HDAC2*, *p* = 0.0098; *HDAC11*, *p* = 9.53e−05) (Figures 3A,D; Figures 4A,C; Table 2). The transcriptional level of *HDAC1* (*p* = 5.06e−57), but not *HDAC2* (*p* = 0.64) and *HDAC11* (*p* = 0.058), significantly varied with the *1p/19q* mutation (Figures 4D,E).

Given that low pathological grade, *IDH1* mutant, and *1p/19q* codeletion were favorable prognostic factors, the fact that *HDAC1* overexpressed in the WHO III and WHO IV group (Figure 3A), in the *IDH1* wildtype group (Figure 4A), and the *1p/19q* non-codeletion (Figure 4D) implied that *HDAC1* tended to be a detrimental prognostic biomarker in glioma (Lapointe et al., 2018). It also suggested the role of *HDAC11* as a protective factor concerning its opposite expression pattern relative to *HDAC1* (Figures 3D, 4C,F). However, *HDAC2* expressed the most in the WHO III group but the least in the WHO IV group, indicating a baffling part *HDAC2* played in the glioma progression (Figure 3A). It was still reasonable that *HDAC2* was involved with the *IDH1* mutation (Figure 4A).

Consistent with the earlier results and assumption, the OS-oriented survival analysis in both the GEPIA (*n* = 676) and the CGGA (*n* = 404) showed that *HDAC1* overexpression (GEPIA, HR:3.9, *p* < 0.0001; CGGA, *p* < 0.0001) brought risks to glioma patients (Figures 5A, 6A), while *HDAC11* served as a favorable prognostic indicator (GEPIA, HR:0.37, *p* = 2.1e−14; CGGA, *p* < 0.0001) (Figures 5C, 6C). Although *HDAC2* (HR:1.3, *p* = 0.024) significantly distinguished the group with poorer prognosis in GEPIA (Figure 5A), it failed to impose any disadvantages to the *HDAC2*-overexpressed group (*p* = 0.39) when validated (Figure 6A). Moreover, *HDAC1* (HR:3.0, *p* < 0.0001) acted as a hazard predictive biomarker, and *HDAC11* (HR:0.5, *p* = 4.8e−08) was still a protective factor in the DFS analysis (Figures 7A,C). The relatively low mutation ratio of *HDAC1* and *HDAC11* probably suggested the stability of the genes (Figure 8A).

In contrast to less efficient discrimination of *HDAC2* and *HDAC11*, the AUC value of *HDAC1* over 0.7 throughout the time points manifested that *HDAC1* was identified to be a promising prognostic biomarker for glioma (Figures 9A–C; Table 2). It was found recently that knockdown of *HDAC1* with siRNA reduced LN18 GBM cell proliferation, leaving cell viability unaffected (Was et al., 2019). The result that simultaneous inhibition of *HDAC1* and *HDAC2* led to a significant drop in GBM cell proliferation synergistically suggested an efficient combination anticancer strategy (Was et al., 2019). More in-depth research found the involvement of *HDAC1* together with *HDAC2* in the regulation of a transcription factor *c-Myc* (Nguyen et al., 2020). The selective and broad inhibition of *HDAC1* and *HDAC2* disrupted *c-Myc* regulation on aerobic glycolysis enhancing oxidative metabolism, followed by peroxisome proliferator-activated receptor γ coactivator1 α (PGC1α) and peroxisome proliferator-activated receptor δ (PPARδ), thereby extending overall survival of patient-derived xenograft (Nguyen et al., 2020). The additional mechanism of *HDAC1*-involved invasive and proliferative phenotype in GBM cells could be attributed to the interaction between *HDAC1* and phosphorylated special AT-rich sequence-binding protein 1 (SATB1) (Han et al., 2013).

It also exposed the moderate relationship between *HDAC1* expression and neutrophil infiltration (r = 0.60, *p* = 2.88e-47) in LGG, as well as CD4^+^ T cell infiltration (r = 0.52, *p* = 3.96e-35) (Figure 9D). Although the studies on how the *HDAC1* expression predicted immune infiltration in LGG were urgently needed, it was demonstrated that IFN-β silenced interleukin-8 (IL-8) transcription by increasing *HDAC1* expression level in GBM cells (Nozell et al., 2006). Given that IL-8 was one of the chemokines that recruited migrating neutrophils, impaired IL-8 release likely resulted in reduced neutrophil infiltration, which might explain the absence of relation between *HDAC1* and neutrophil infiltration in the GBM.

The components of the final *HDAC1*-related signature could be the reason why the separability of the signature exhibited robust efficiency (1-year OS AUC = 0.80, 3-years OS AUC = 0.84, 5-years OS AUC = 0.829, 10-years OS AUC = 0.882) (Figures 10D,E). In the signature, *HDAC1* (HR:1.4938) and *RBL1* (HR:1.7148) were deemed a hazard, while *RUNX1T1* (HR:0.7349), *FKBP3* (HR:0.6382), and *PHF21A* (HR:0.4956) promoted survival possibilities for glioma patients (Figure 10A). The *HDAC1*-related signature was adjusted as an independent risk indicator and eventually validated both internally and externally showing reliable discrimination for glioma prognosis prediction (Supplementary Figure S3; Figures 10D–F; Figures 11A,B).

Of note, *HDAC1* was transcriptionally regulated by the nuclear factor of activated T cell (NFAT), which played a role in glioma stem cell growth and mesenchymal transition (Song et al., 2020). Retinoblastoma transcriptional corepressor like 1 (RBL1), known for its modulation in the G1/S cell cycle, behaved oppositely and functioned as a tumor suppressor in a GBM model, conflicts of which could come from either the species’ differences or some other regulations unidentified (Naert et al., 2020). RUNX1 partner transcriptional co-repressor 1 (RUNX1T1) earned prestige for its fusion with Runt-related transcription factor 1 (RUNX1) in acute myeloid leukemia (Beghini, 2019). Related to HDAC class I signaling, FK506 Binding Protein 3 (FKBP3) regulated *HDAC2* expression contributing to the drug resistance in tumor cells (Tong et al., 2019). PHD finger protein 21A (PHF21A) was mainly studied in neurodevelopmental disorders (Vallianatos and Iwase, 2015). Though the research on *RUNX1T1*, *FKBP3*, and *PH21A* in glioma was definite, the signature was annotated as “chromatin binding” by the functional enrichment analysis (Figure 10E).

However, the prognostic *HDAC1* per se and the *HDAC1*-related gene signature were yet to be employed and verified universally. Considering some potential barriers when promoting sequencing techniques in clinical oncology testing, it would require more time for the real arrival of the molecular prognostic biomarker era. In addition, it remains a question to be solved regarding how *HDAC1* was involved with the glioma progression and the modulation of immune infiltration in glioma. Acetylation and deacetylation were assumed to stand downstream in the HDAC-involved glioma modulation and thereby possibly influence glioma prognosis, which also warranted large-scale data to testify in the future. How epigenetics like methylation affected glioma through regulation of the HDACs remains another interesting topic.

## Conclusion

Conclusively, the comprehensive analysis of the HDAC family exposed that the HDAC family components were of prognostic significance for glioma. Overexpressed *HDAC1* in glioma positively altered its expression with glioma grade, *IDH1* wildtype, and *1p/19q* non-codeletion, while *HDAC11* was downregulated and acted oppositely compared to *HDAC1*. The majority of the HDAC family significantly correlated with the grade, *IDH1* mutation, and *1p/19q* codeletion in glioma. *HDAC1* was manifested to serve as a prognostic biomarker for glioma, and an indicator for neutrophil and CD4^+^ T cell infiltration in LGG. Ultimately, the *HDAC1*-centered signature composed of the *HDAC1*-related genes was developed and validated for precise prognosis prediction in glioma. It was tempting to speculate an optimistic future for glioma not only by implementing *HDAC1* and other HDACs as prognostic biomarkers but also by targeting *HDAC1* and its closely related genes.

## Data Availability

The original contributions presented in the study are included in the article/Supplementary Material, further inquiries can be directed to the corresponding author.

## Supplementary Material

Supplementary Figure S1 LASSO regression of the HDAC1-related genes. The trajectory plot on the left panel was for independent variables sourced from the HDAC1-related genes. The plot on the right panel showed that each lambda and confidence interval. LASSO, least absolute shrinkage, and selection operator.

Supplementary Figure S2 Candidate HDAC1-related genes for the signature under proportional hazard assumption.

Supplementary Figure S3 The HDAC1-related signature adjusted to be an independent risk indicator.

Supplementary Table S1 HDAC1-related genes collected from STRING and GeneMANIA.

Supplementary Table S2 HDAC1-related genes with significance in univariate and multivariate Cox regression analyses.

## Abbreviations

AUC, area under the curve ; CNS, central nervous system; CI, confidence interval; DFS, disease-free survival; FC, fold change; FKBP3, FK506 binding protein 3; GEPIA, gene expression profiling interactive analysis; GTEx, genotype-tissue expression project; GBM, glioblastoma multiforme; HR, hazard ratio; HAT, histone acetyltransferase; HDAC, histone deacetylase; IDH1, isocitrate dehydrogenase 1; KM, Kaplan-Meier; LGG, lower-grade glioma; MDSC, myeloid-derived suppressive cell; NFAT, nuclear factor of activated T cell; OS, overall survival; PPARδ, peroxisome proliferator-activated receptor δ; PGC1α, peroxisome proliferator-activated receptor γ coactivator1 α; PHF21A, PHD finger protein 21A; PH, proportional hazards; ROC, receiver operating characteristic; Tregs, regulatory T cells; RBL1, retinoblastoma transcriptional corepressor like 1; RUNX1, runt-related transcription factor 1; RUNX1T1, RUNX1 partner transcriptional co-repressor 1; STRING, search tool for the retrieval of interacting genes; SATB1, special AT-rich sequence-binding protein 1; TCGA, the cancer genome atlas; LASSO, the least absolute shrinkage and selection operator; TIMER, tumor immune estimation resource.
