# Supplementary figures and images for: Comprehensive Analysis of HDAC Family Identifies HDAC1 as a Prognostic and Immune Infiltration Indicator and HDAC1-Related Signature for Prognosis in Glioma
Source: Front Mol Biosci. 2021 Sep 1;8:720020. doi: 10.3389/fmolb.2021.720020 (PMC8442956; doi:10.3389/fmolb.2021.720020)

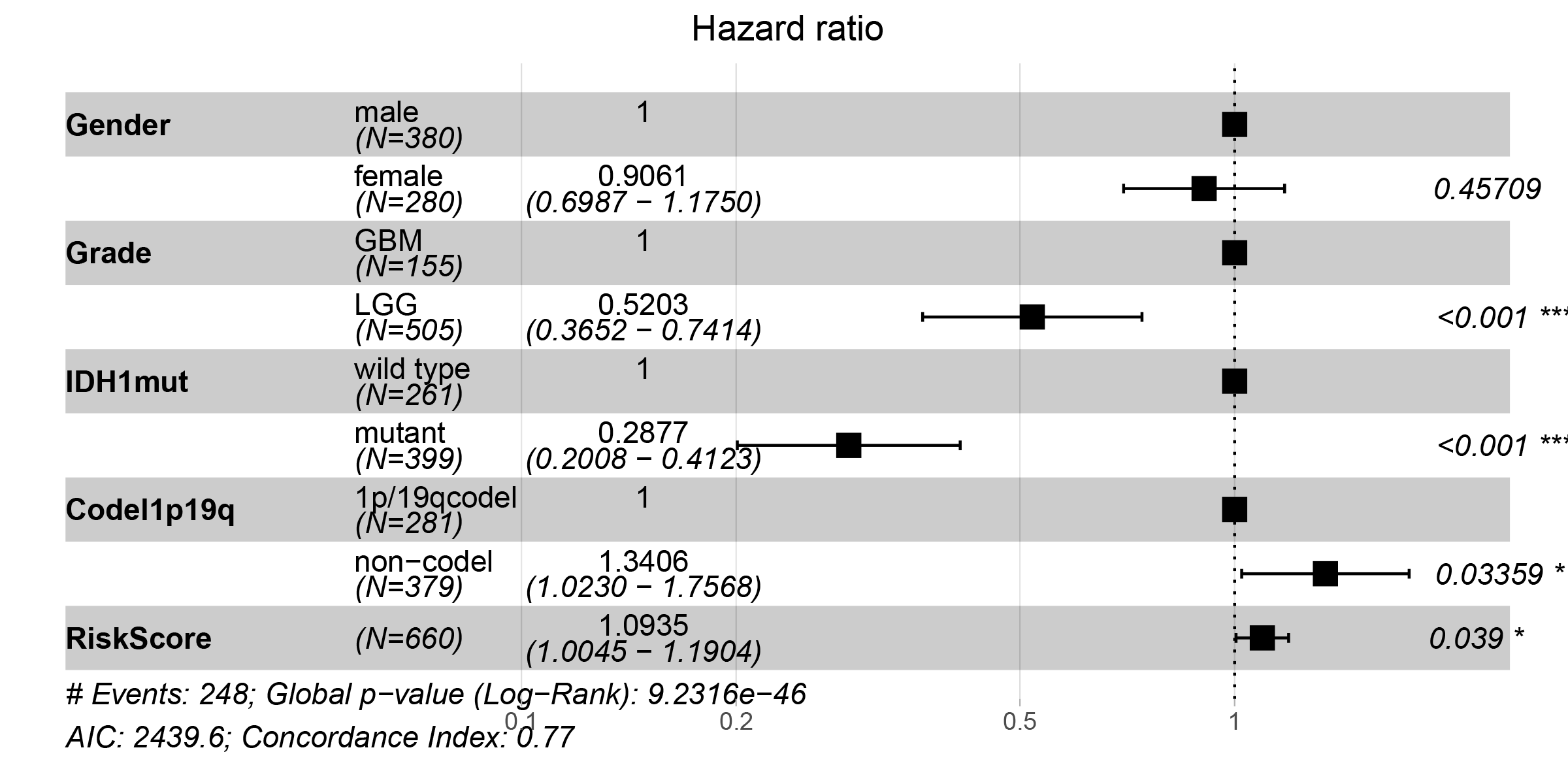

Supplement: Supplementary file 2 [file image3.tif]

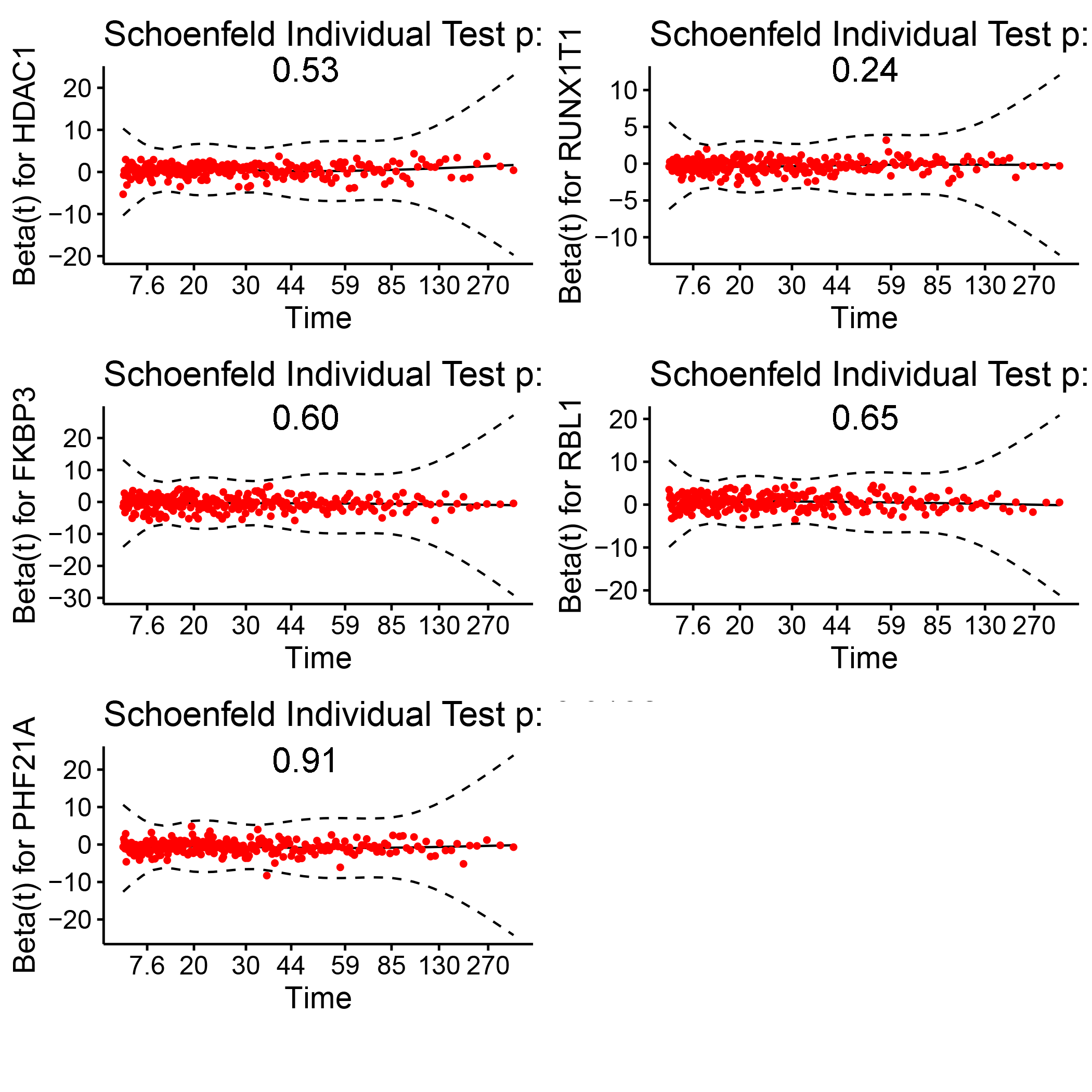

Supplement: Supplementary file 3 [file image2.tif]

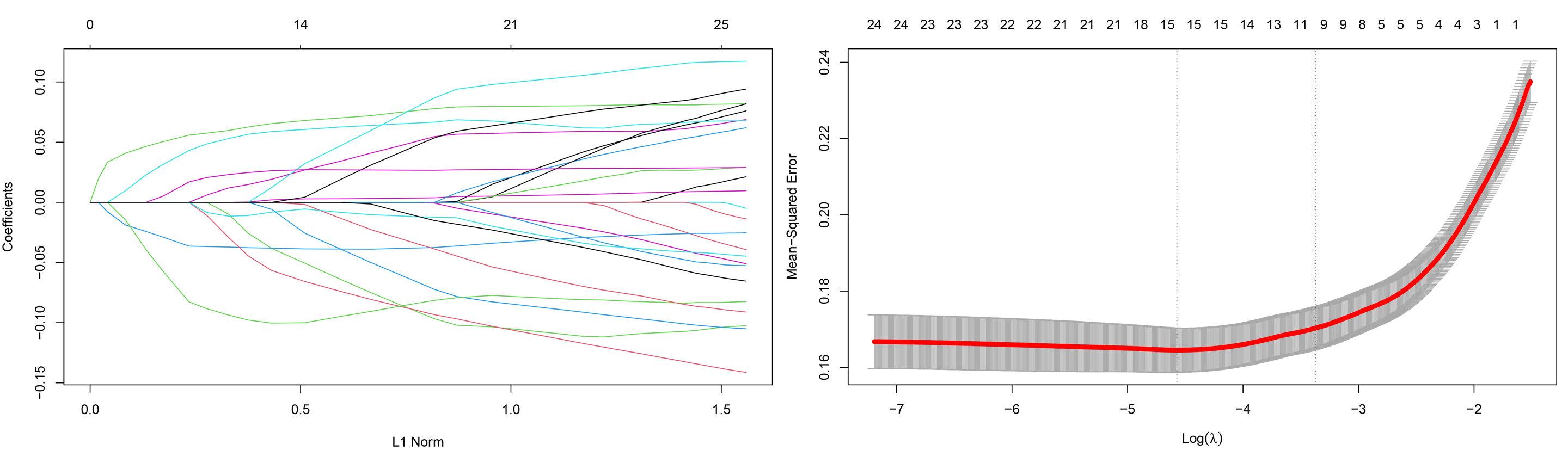

Supplement: Supplementary file 4 [file image1.tif]
