# Supplementary material for: Comprehensive Analysis of HDAC Family Identifies HDAC1 as a Prognostic and Immune Infiltration Indicator and HDAC1-Related Signature for Prognosis in Glioma
Source: Front Mol Biosci. 2021 Sep 1;8:720020. doi: 10.3389/fmolb.2021.720020 (PMC8442956; doi:10.3389/fmolb.2021.720020)
Supplement: Supplementary file 5 [file table3.docx]

|  | GEO cohort | | |  | TCGA cohort | | |
| --- | --- | --- | --- | --- | --- | --- | --- |
|  | n = 221 | | |  | n = 660 | | |
| **Age** (years) | median (50.34) |  |  |  | median (46.64) |  |  |
|  | age > 65 | 43 | 19.46% |  | age > 65 | 94 | 14.24% |
|  | age ≤ 65 | 178 | 80.54% |  | age ≤ 65 | 565 | 85.61% |
| **Gender** |  |  |  |  |  |  |  |
|  | male | 153 | 69.23% |  | male | 380 | 57.58% |
|  | female | 68 | 30.77% |  | female | 280 | 42.42% |
| **Grade** |  |  |  |  |  |  |  |
|  | LGG | 93 | 42.08% |  | LGG | 505 | 76.52% |
|  | GBM | 128 | 57.92% |  | GBM | 155 | 23.48% |
| ***IDH1* mutation** | |  |  |  |  |  |  |
|  | wild type | 140 | 63.35% |  | wild type | 261 | 39.55% |
|  | mutant | 81 | 36.65% |  | mutant | 399 | 60.45% |
| **1p/19q codeletion** | |  |  |  |  |  |  |
|  | non-codel | 183 | 82.81% |  | non-codel | 379 | 57.42% |
|  | 1p/19qcodel | 38 | 17.19% |  | 1p/19qcodel | 281 | 42.58% |
| **Chemotherapy** |  |  |  |  |  |  |  |
|  | Yes | 84 | 38.01% |  | Yes | 459 | 69.55% |
|  | No | 137 | 61.99% |  | No | 201 | 30.45% |
| **Radiotherapy** |  |  |  |  |  |  | 0.00% |
|  | Yes | 154 | 69.68% |  | Yes | 478 | 72.42% |
|  | No | 67 | 30.32% |  | No | 182 | 27.58% |
| **Overall Survival** (months) | |  |  |  |  |  |  |
|  | OS < 12 | 100 | 45.25% |  | OS < 12 | 181 | 27.42% |
|  | OS ≥ 12 | 57 | 25.79% |  | OS ≥ 12 | 297 | 45.00% |
|  | OS ≥ 36 | 22 | 9.95% |  | OS ≥ 36 | 105 | 15.91% |
|  | OS ≥ 60 | 30 | 13.57% |  | OS ≥ 60 | 59 | 8.94% |
|  | OS ≥ 120 | 12 | 5.43% |  | OS ≥ 120 | 18 | 2.73% |

**Supplementary Table 3.**  The clinical baseline table of the TCGA and GEO cohort
